# Assessment of the Applicability of Selected Data Mining Techniques for the Classification of Mortars Containing Recycled Aggregate

**DOI:** 10.3390/ma15228111

**Published:** 2022-11-16

**Authors:** Bernardeta Dębska

**Affiliations:** Department of Building Engineering, Rzeszow University of Technology, ul. Poznańska 2, 35-959 Rzeszów, Poland; bdebska@prz.edu.pl; Tel.: +48-178-652-077

**Keywords:** epoxy mortars, polyester mortars, cement mortars, waste materials, discriminant analysis, decision trees, cluster analysis, mechanical properties, physical properties, sustainability

## Abstract

The article contains the results of selected tests of physical and mechanical properties of mortars differentiated in terms of the binder used: cement, epoxy, epoxy modified with PET waste glycolysate and polyester. Each type of mortar was modified by partial (0–20% vol.) substitution of sand with an agglomerate made from waste polyethylene. The obtained results were used to build a database of mortar properties, which was then analyzed with the use of three different techniques of knowledge extraction from databases, i.e., cluster analysis, decision trees and discriminant analysis. The average results of the properties tested were compared, taking into account the type of mortar, indicating those with the most favorable parameters. The possibilities and correctness of mortar classification with the use of the indicated “data mining” methods were compared. The results obtained confirmed that it is possible to successfully apply these methods to the classification of construction mortars and then to propose mortars with such a composition that will guarantee that the composite will have the expected properties. Both the presented method of plastic waste management and the proposed statistical approach are in line with the assumptions of the currently important concept of sustainable development in construction.

## 1. Introduction

A key trend determining the future of construction is ecology-related issues. Ecological construction is aimed at reducing the amount of waste and minimizing the energy consumption related to the production and transport of building materials. The term “sustainable construction” refers to human activities related to the design and construction of facilities that should be carried out on an equal footing with care for the natural environment and economical use of raw materials at all stages of the building life cycle. Waste generation should be minimized and, if it does occur, it should be treated as a source of secondary raw materials. For this reason, an additional element added to the process of designing innovative building materials is the study of the possibility of recycling waste and reproducing a material with better or equally good properties. This approach allows for the introduction of a closed circuit (LCA—Life Cycle Assessment) in construction, which is a process to assess the effects that a given product (material) has on the environment throughout its life, by increasing the effective use of resources and reducing the environmental burden [1,2]. At the same time, it will allow one to partially solve the problem of the currently growing shortages of natural resources and the introduction of waste in its place. This approach is extremely important because traditional raw materials, such as sand, are becoming scarce, and the costs of their extraction, processing, transport and distribution are constantly increasing [3,4,5].

Aggregates are currently one of the most desirable materials in construction. In 2019, the United Nations made the extraction of aggregates (including sand and gravel) an issue of international protection. It indicated the need to search for a common answer to dangerous interrelated trends such as unrestrained consumerism and the drastic shrinkage of natural resources. It was stated that demand should be reduced and substitutes should be found because even such a common raw material as sand may soon be lacking, especially in the context of the fact that not all types of sand are suitable for construction purposes. Although inconspicuous, this raw material is the foundation of modern civilization, and its annual consumption currently reaches billions of tons. The widespread consumption of natural aggregates and the progressive degradation of the environment force the search for alternative aggregates. It seems that the reuse of waste as aggregate for concrete may bring positive effects both in terms of the possibility of recycling waste and in terms of the protection of natural resources [6,7,8]. It is particularly important to manage plastic waste in this way, as it is known that the world’s dependence on plastic is still growing [8,9,10]. Researchers estimate that by 2050 the total amount of plastic waste in the world could reach more than 25 billion tons. In this group of wastes, polyolefins have the highest share, including polyethylene (PE) and polyethylene terephthalate (PET). The positive effects of modifying the composition of concrete and mortars with waste of these plastics were noted, among others, by the authors of works [1,6,11,12,13,14].

When planning the use of waste in the construction material design process, it should be remembered that the use of waste is possible only under the condition that its addition does not significantly reduce the quality of construction products despite the replacement natural resources (e.g., aggregate) with recycled materials. Therefore, the development of a new recipe requires a lot of research and analysis. Proper planning of the experiment enables one to obtain useful and complete information about the tested material and at the same time results in limiting the financial outlays generated by research. Practical benefits can be derived from the information confirmed experimentally, so it is worth paying attention to both the proper planning of research and the search for methods of obtaining knowledge from existing databases (data mining) that will enable the maximum effective use of all the results of previously conducted research. Such tools include techniques of knowledge mining from databases, available, e.g., in the Statistica program [15].

The database is a selected and structured set of information on the material, technology, its properties, and areas of use. The database can be presented as a matrix composed of rows and columns that are filled with not only numbers, but also quality characteristics, names, etc. The composite database consists of input data that inform about what is known about a given material and its execution, and output data that can be the subject of analysis, classification, prediction, etc. The creation of a laboratory database and the use of knowledge mining techniques from these databases using various techniques (discriminant analysis, decision trees, cluster analysis) allows for the development of models that will allow for prediction of features in future materials, without the need to perform tests. This fact is particularly important in the case of building composites, for which, in order to determine their specific properties, a series of destructive tests must be performed, which is laborious and generates costs. This approach is also consistent with the principles of sustainable development. Exploratory methods also enable the classification of composites described by a given set of parameters (features, input data) into an appropriate group. The more composites tested using different types of waste materials and with different matrices, the larger the database, the wider the analytical possibilities and the richer the knowledge about these materials. Such databases are most often built with an incremental method, i.e., each designed and tested composite is entered as a new row into the existing database. 

Unfortunately, these techniques are not used very often in the context of building materials. Most of the information in this field is related to the need to predict the mechanical strength of concrete. The imperfection of widely used empirical and statistical models for this purpose, such as linear and non-linear regression, forced the necessity to use alternative methods in the form of machine learning (ML) models, including artificial neural networks, decision trees, or evolution algorithms [16,17,18].

Scientists often use artificial neural networks (ANN) in their work. In this case, the root mean square error (RMSE) is most often used to assess the forecasting ability and accuracy. This is the standard way to measure model error in predicting quantitative data. The root mean square error (RMSE) is the square root of the root mean square of all errors. The use of RMSE is very common and is considered an excellent general-purpose error metric for numerical predictions. Article [19] predicted the compressive strength of high performance concrete with high volume ground granulated blast furnace slag. The database included 152 cases. It was observed that the artificial neural network developed allows one to predict strength with the RMSE error at the level of 3.4 MPa (0.03%). In [20], machine learning algorithms were used to assess the interlayer bond between a concrete repair mortar and a component being repaired. To predict the adhesion value (pull-off test), the authors used an artificial neural network, a support vector machine, and a random forest algorithm based on 338 samples. They obtained a very good RMSE of 0.341. In turn, in [21] an extensive simulation program was used to fit an ANN model to predict the compressive strength of concrete containing ground granulated blast furnace slag (GGBFS). For this purpose, an experimental database with 595 samples was created on the basis of data from the literature. The RMSE was obtained at the level of 3.803 MPa. The authors of work [22] presented the possibility of using artificial intelligence (ANN) to predict the depth of concrete consumption modified with high calcium fly ash. According to the authors, the model developed on the basis of 216 data is highly useful (RMSE = 0.06%), especially in the context of destructive tests, and can help in the design of a cement fly ash composite with high calcium content with increased durability.

Neural networks work well when there is a lot of data. The database discussed in this publication contains 120 cases and is under development. After completing this process, it will be possible to check the classification correctness for a system built with the use of ANN.

The authors of article [23] described a comparison of various statistical methods (including decision tree, random forest, K-nearest neighbor) to predict the strength characteristics of reinforced steel fiber concrete. Forecasting the mechanical properties of concretes obtained with the use of alternative aggregates is described in, among other works [24,25,26,27]. The authors of article [24] used three tree-based models: one individual model (regression tree (RT)) and two team models (random forest (RF) and gradient-enhanced regression tree (GBRT)). Additionally, they developed user-friendly graphical interfaces to make it easier for practicing engineers to apply AI models to the design of concrete mix. In the remaining work, model building was also based on artificial neural networks (ANN) and decision trees.

The authors of work [28] described the hierarchical use of an agglomeration clustering algorithm (cluster analysis) to group resin composites including fibers into classes according to their closeness in the k space of the geometric features of the fibers. The division into nine classes shown allows for a detailed analysis of the fiber morphology. According to the authors of article [29], cluster analysis can help in a quick assessment of moisture transport characteristics for a given microstructure of two-phase fiber-reinforced polymer composites with any proportion of shape and volume. The cluster analysis was also successfully used by the authors of [30] to classify concretes containing various types of aggregates.

This article presents the results of research on four types of building mortars differentiated in terms of the binder used, they were: epoxy mortars, epoxy mortars modified with poly (ethylene terephthalate) glycolysate, polyester, and cement mortars. These composites were obtained with partial substitution (0–20% vol.) of natural aggregate by agglomerates made of waste polyethylene bags (PE). The mortars were tested for strength parameters and the bulk density and water absorption were also determined. The results obtained were recorded in a laboratory database and then the mean values of the individual parameters, determined for each type of composite, were calculated and compared. Subsequently, the data were analyzed using three different techniques of knowledge mining from databases, i.e., discriminant analysis, decision trees, and cluster analysis. The results obtained confirmed that the selected “data mining” methods can be successfully applied to the classification of building mortars. The computational experiments carried out on the test samples showed the correctness of the classification for data other than those on which the systems learned. Both the presented method of plastic waste management and the proposed statistical approach are in line with the assumptions of the currently important concept of sustainable development in construction.

## 2. Materials and Methods

### 2.1. Materials

Four types of mortars were obtained, differing in the type of binder. The first type of mortar was based on a cement binder, the characteristics of which are presented in Table 1. In three subsequent cases, a chemically hardened resin was used as a binder: polyester resin (Polimal 109, CIECH Sarzyna S.A., Nowa Sarzyna, Poland), epoxy resin (Epidian 5, CIECH Sarzyna S.A.), epoxy resin (Epidian 5, CIECH Sarzyna S.A.) modified with glycolysate obtained on the basis of propylene glycol and poly(ethylene terephthalate) (PET) waste. The selected properties of the resin binders are summarized in Table 2. Modification of epoxy mortar with PET glycolysate consisted of a partial substitution (9 wt%) by glycolysate. The glycolysate was in the form of semi-solid wax with a density of 1.3 g/cm^3^, a melting point in the range 78 to 82 °C, and a hydroxyl number of 515 mg KOH/g. Before making the mortar, the resin-glycolysate composition was heated for 1 h at a temperature of 85 °C and then stored in the laboratory until the temperature reached 21 ± 2 °C. The resin mortars were hardened with the appropriate hardeners for a given resin (triethylenetramine (Z-1) for epoxy and Metox-50 for polyester), the typical characteristics of which are presented in Table 3. 

**Figure 1:**
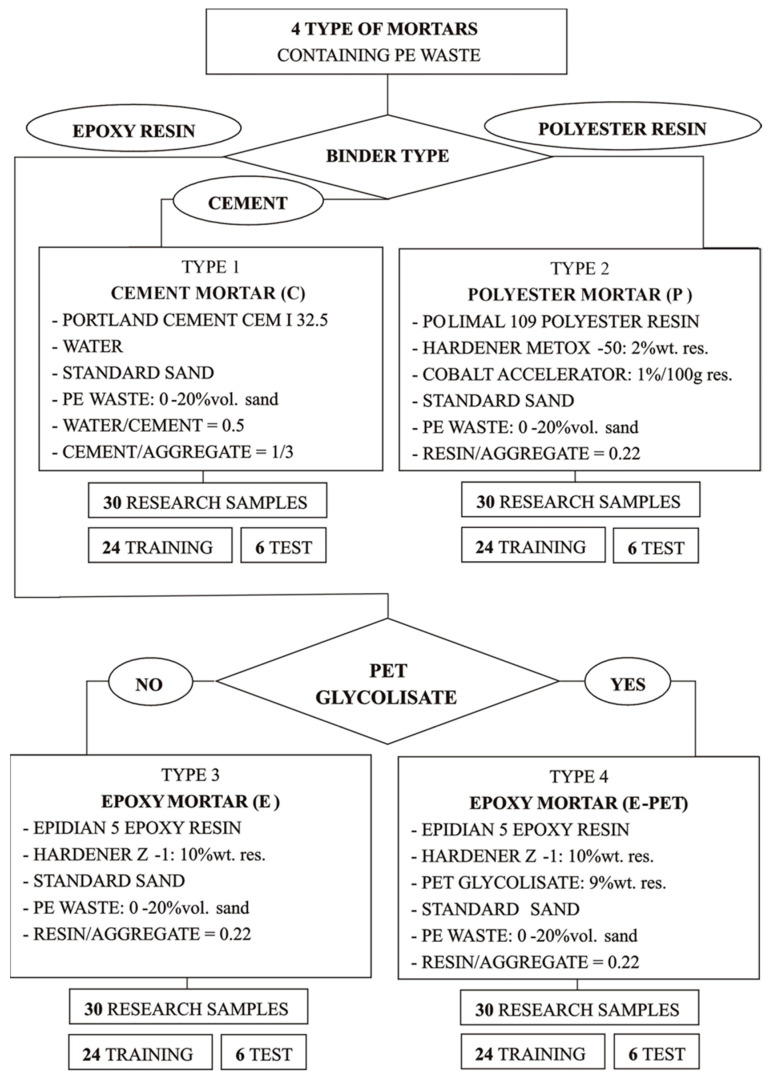
The method of obtaining and division of mortars.

In the case of polyester resin, additionally a 1% cobalt accelerator was used. The basic aggregate in all types of mortar was standard quartz sand with a grain size of 0–2 mm. It was partially replaced (0–20% by volume) with an agglomerate obtained on the basis of waste polyethylene bags. Sand substitution took place proportionally in each fraction. The scheme for obtaining and division of mortars is presented in Figure 1. 

For each type of mortar, 30 samples with dimensions of 40 mm × 40 mm × 160 mm were prepared, which were then used to determine the strength parameters (flexural and compressive strength), bulk density and water absorption. From 30 experimental data (separately for a given type of mortar), 6 test data are randomly selected. The remaining results constitute the training data set.

### 2.2. Methods

#### 2.2.1. Physico-Mechanical Research Methods

- Flexural and compressive strength

The flexural strength and compressive strength tests were carried out in strength machines, respectively: Cometech Testing Machines Co., Taichung, Taiwan, with a maximum load capacity of 50 kN and MATEST S.p.A., Arcore, Italy, with a maximum load capacity of 1500 kN. The tests were carried out in accordance with the PN–EN 196-1: 2016 standard [31]. In the flexural strength test, the sample was loaded at a speed of 0.25 mm/min (resin mortars) or 1.00 mm/min (cement mortars). Halves of the bars remaining after the flexural strength test were used for the compressive strength test. The sample was loaded at a speed of 2.4 kN/s.

- Bulk density

The bulk density determination was carried out on samples with dimensions of 40 mm × 40 mm × 160 mm. The mass of the samples dried to constant mass was determined on technical scales. The volume of the samples was calculated from their dimensions. The value of the bulk density was determined according to Formula (1):

(1) $$b_{d}=\frac{m}{V}$$

where

*b_d_*—bulk density, g/cm^3^,
*m*—sample weight, g,
*V*—sample volume, cm^3^.

- Absorptivity

The determination of the absorbability of cement mortars was carried out on samples with dimensions of 40 mm × 40 mm × 160 mm, and for resin mortars on samples with dimensions of 60 mm × 60 mm × 5 mm. The mass of samples dried to constant mass was determined on technical scales. Then, they were subjected to a process of soaking with water until a constant weight was obtained. The water absorption was calculated on the basis of Formula (2):

(2) $$Abs=\frac{m_{n}-m_{s}}{m_{s}}\cdot 100\%$$

where

*Abs*—absorptivity, %,
*m_n_*—mass of the sample saturated with water, g,
*m_s_*—mass of the sample dried to constant mass, g.

#### 2.2.2. Statistical Methods

Thanks to the associative representation of experimental data (input data describing the composition of the composite with output data defining the properties), it is possible to generate a variety of knowledge about the studied process, and then generalize it, transfer it to new research objects, and thus verify it and look for optimal solutions.

From the group of problems that can be solved using “data mining” methods, in the case of the obtained database of mortars modified with polyethylene waste, a model classification was selected, including methods such as discriminant analysis, decision trees and cluster analysis. The use of more than one “data mining” method to solve the problem is justified because, inter alia, obtaining convergent solutions using various algorithms from the “data mining” group can be treated as a factor that strongly confirms the formulated conclusions.

All statistical analyses were performed in the Statistica 12 program, using the Basic Statistics and Tables modules, as well as *Multidimensional Exploratory Techniques*.

- Discriminant analysis

Discriminant analysis is one of the statistical methods used to resolve the variables that best divide a given set of cases into groups that occur naturally in it. These methods lead to the finding of a classification rule, class characteristics, or class separation functions on the basis of the training set, that is, containing objects with known class affiliation (classification with the teacher) [32].

There are two main stages in discriminant analysis:

1. The learning stage (model building), in which classification rules are created based on the research results stored in the database.

The canonical discriminant functions that separate the studied groups are determined. In the case of differences between groups, each of them can be treated as a cloud of points in space with the axes being discriminating variables. The classification process involves creating one or more functions that classify the analyzed cases into appropriate groups. There are as many functions as there are groups, and they are used to decide which group most likely represents a given case.

2. The classification stage, in which a set of objects, the membership of which is unknown, is classified based on previously identified class characteristics.

- Decision trees (classification)

The techniques used in decision trees have much in common with traditional techniques of discriminant analysis methods but are easy to graphically represent, making them easier to interpret than purely numerical results. Decision trees are a family of statistical methods that use diagrams to sequentially divide the investigated data set into classes with similar properties [24,33,34].

The decision tree consists of the following.

➢ Root, the beginning of the recursive partitioning process;
➢ The branches lead from the root to the next nodes;
➢ Node, a place where a certain condition concerning a given observation is checked, and on its basis one of the branches leading to the next, lower node is selected;
➢ Parent of nodes, the place from which branches directed to subsequent nodes emerge;
➢ Descendants, nodes connected to the parent;
➢ Leaf—terminal node terminating the path of inference in which there is no subdivision of data (no children). It contains information about the assignment of data in a subspace to a specific class.

The purpose of creating a classification tree is to obtain a model that will allow for the classification of future observations, i.e., those with unknown membership of a given class. The tree model allows not only for prediction but also for the description and presentation of patterns in the surveyed community. At each stage of the tree construction, all the variables (predictors) are analyzed, and the one that provides the best division of the node is finally selected, i.e., the most homogeneous subsets can be obtained. In each subsequent node, a different independent variable can be used for the analysis, and the division in a given node is made only on the basis of the training sample vectors that reached the given node. This division should be such that the diversity of the parts obtained from the data set reaching the descendants is as small as possible [35,36]. Classification, that is, determining the class of a new example (object) with a decision tree, which consists of moving from the root to one of the leaves.

- Cluster analysis

Cluster analysis aims at establishing the relationship between data, defining the structure of the data set, and, consequently, the classification of multidimensional objects (multivalued knowledge associations). The purpose of the classification is to indicate subsets in the entire data set that group the most similar objects (clusters). The clusters can be of different sizes, have a different shape, and can be differently arranged in relation to each other in the feature space. Cluster analysis is therefore an important tool for interpreting multidimensional data that include objects, features, and properties. An object is any real object, such as a sample, e.g., mortar or manufacturing process. The object is characterized by a set of features. The feature is a numerical variable, and in this article these are the results of physical and mechanical tests: flexural and compressive strength, water absorption, and volume density. These data can best be described by an X matrix (n × p) matrix, which contains a row for each object and a column for each property. Each object then has a corresponding point in the p-dimensional feature space.

Cluster analysis was performed in two stages, using the following techniques, respectively:

(a) Hierarchical agglomeration—hierarchical cluster analysis.

Hierarchical analysis produces a sequence of nested clusters that can be represented as a dendrogram. At the base of the dendrogram are all the elements of the data set. The vertical axis gives the degree of cluster diversity. This value is generally standardized in the range [0, 1]. The horizontal lines connecting the clusters define the level of dissimilarity for which the agglomeration of component clusters took place. There are two basic types of algorithm for obtaining this sequence—agglomeration and division. Agglomerating algorithms construct a dendrogram starting from the leaves (individual elements of a set) towards the root (whole data). In each step, the two closest clusters are combined. The basis for their selection is the criterion of similarity of clusters. Usually, the criterion of the minimum, average, or maximum distance between the elements of two clusters is used. The distance between the objects analyzed is considered a measure of their similarity and can be defined, inter alia, as the Euclidean distance, the Chebyshev distance, city distance (Manhattan, City block) [37]. In the presented research, the cluster analysis is based on the calculation of distances using the Euclidean method because the operations were performed on an experimental data which are of a continuous nature. The purpose of data interpretation was to search for clusters containing similar objects and to study the relationship between a set of features and a set of properties.

(b) Iterative division in the light of the selected criterion, e.g., minimizing the dispersion within a cluster—cluster analysis using the k-means method.

As a result of the application of the k-means method, the data are horizontally divided into k disjoint clusters. The measure of spread within a cluster is the sum of the squares of the distance of individual data vectors from the cluster center, which is the mean vector. The sum of these measures across all clusters is called the mean square error for the system of k clusters. The algorithm starts with a random division of the data set into clusters. In subsequent iterations, the vectors are shifted between the clusters, and the cluster centers are recalculated. The algorithms task is to divide data into k clusters that minimize the error for the entire system. It is manifested by the lack of further vector shifts [38].

## 3. Results and Discussion

**Figure 2:**
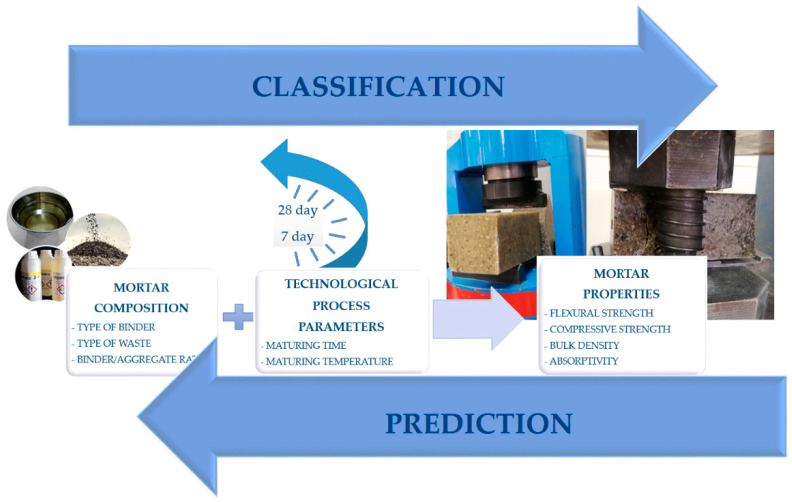
Diagram of the technological process of obtaining mortars.

During the laboratory experiments, the experimental data were collected, which became the basis of the computer database. This database was created incrementally, each new sample test results were entered into the database, expanding it with new data. The data set created a matrix with n = 120 cases (mortar types) and m = 4 variables (mortar properties). When creating the database, a specific formalism was used to represent knowledge about the technological process (knowledge association), which can be presented using the general scheme presented in Figure 2.

**Figure 3:**
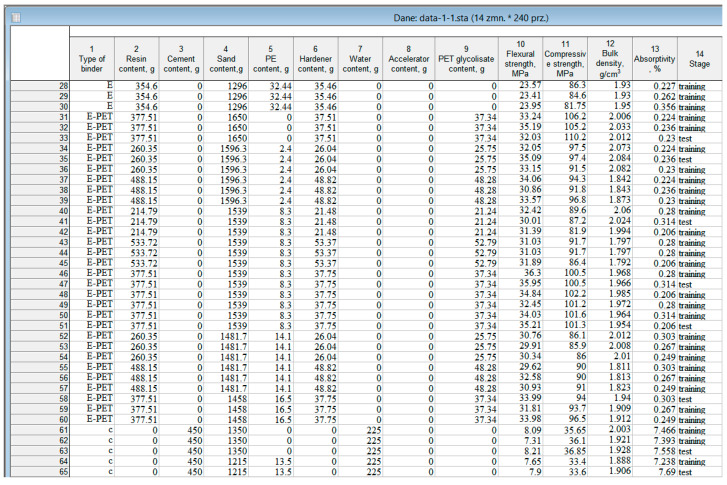
Fragment of the experimental database.

Each such association describes one mortar sample, and the set of all associations constitutes the experimental database. These data are summarized in the form of a table, a fragment of which is shown in Figure 3.

The input table contained 14 columns (Figure 3). The first column contains information on the binder used to obtain the mortars (Type of binder—marked, respectively, as c for cement mortars, E for epoxy mortars, E-PET for epoxy mortars modified with PET waste glycolysate, and P for polyester mortars). The type of binder was the so-called grouping variable that identifies the type of mortar. The contents of columns 2–9 describe the composition of individual mortars. The following columns (10–13) of the table summarize the values of flexural strength, compressive strength, volumetric density, and water absorption determined for the samples prepared. The last column of the table (Stage) contains labels on the basis of which a system will be created and assessed, classifying mortars into four groups (types), distinguished in the first column of the table. This variable is a sample identifier and allows us to distinguish between the sample intended for analysis (Training) and the sample intended for cross-validation (Test), which allows for the assessment of the quality of the classifier.

The table with experimental data was saved as a data sheet in Statistica 12 and used to perform statistical analyses.

### 3.1. Descriptive Statistics

**Figure 4:**
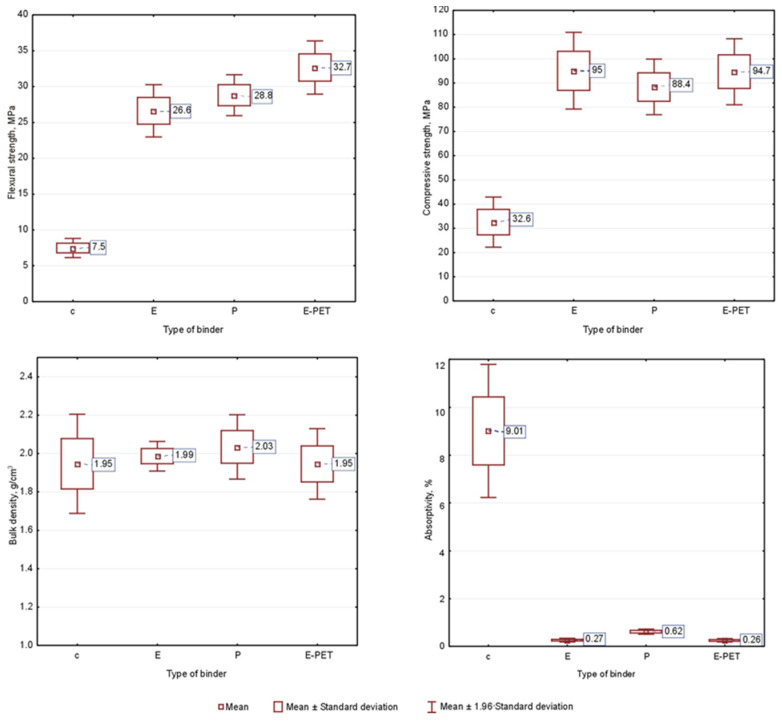
Descriptive statistics of marked properties of mortars.

Data analysis started with the calculation of descriptive statistics in the form of mean values and standard deviation for the properties data obtained within each type of mortar. Box-whisker graphs showing the ranges of the variables studied, generated in the Statistica program, are presented in Figure 4.

From the graphs in Figure 4, it can be seen that there is a differentiation of the properties tested depending on the type of binder (type of mortar).

By definition, cement mortars are characterized by lower strength parameters and much higher water absorption than mortars with a resin matrix. However, in the context of bulk density, no significant differences were observed, the average value of this parameter ranges from 1.95 g/cm^3^ for cement and epoxy mortars modified with PET glycolysate to 2.03 g/cm^3^ for polyester mortars. Resin mortars are characterized by very high flexural and compressive strength. It can be seen that partial substitution of the epoxy resin with PET glycolysate allows for significant improvements in the flexural strength (by approximately 23%), but it has no major impact on the obtained results of compressive strength.

Polyester mortar is characterized by lower compressive strength and more than twice as high water absorption compared to other resin mortars. However, these properties are at a very good level. The proposed modification, consisting of a partial replacement of sand with an agglomerate of waste polyethylene, makes it possible to obtain mortars with favorable physical and mechanical parameters. With a small (up to 10 vol.%) degree of substitution, a slight increase in strength can be observed. A further increase in the amount of waste in composites leads to deterioration of properties, but they remain at a satisfactory level. The dispersion of the results is related to the fact that the data set consisted of mortars characterized by a different degree of sand substitution by the PE waste agglomerate.

### 3.2. Discriminant Analysis

The creation of the diagnostic system was performed using the multidimensional exploration techniques module implemented in the Statistica 12 program. The process was carried out in three steps, on the data set partially shown in Figure 3.

Step 1—Learning. Based on the cases that make up the training data set (96 cases labeled Training in the Stage column), a classifier was built. The analysis of discriminant functions allows us to decide at this stage which variables allow the best way to divide a given set of cases into groups that occur naturally.

The results summarized in Table 4 provide two valuable pieces of information. First of all, the values of the Wilks lambda parameter are close to 0, which shows the very good discriminant power of the currently built model. Second, the parameter of the Wilks’ partial lambda, determines the specific contribution of a given variable to the process of group discrimination. The lowest value of this parameter for flexural strength confirms that this property is the main variable that enables the discrimination of mortars differing in the type of binder. Subsequently, compressive strength and absorptivity make the greatest contribution to overall discrimination, while bulk density contributes the least.

The results of the Chi-square test presented in Table 5 show that the three discriminant functions are statistically significant (*p* < 0.05) and all three can be interpreted. The determination of the canonical discriminant functions was carried out on the basis of the calculated standardized coefficients of these functions, which are summarized in Table 6. From the last row of Table 6 it can be concluded that the first function is responsible for more than 96%, and the second for more than 99% of the explained variability (variance) hidden in the data. Therefore, these two functions are important. The first differentiates mainly cement mortars (the canonical average presented in Table 7, equal to −14.454, is definitely different from the others). Similarly, it can be concluded that the second function mainly distinguishes epoxy and the third polyester mortars.

**Figure 5:**
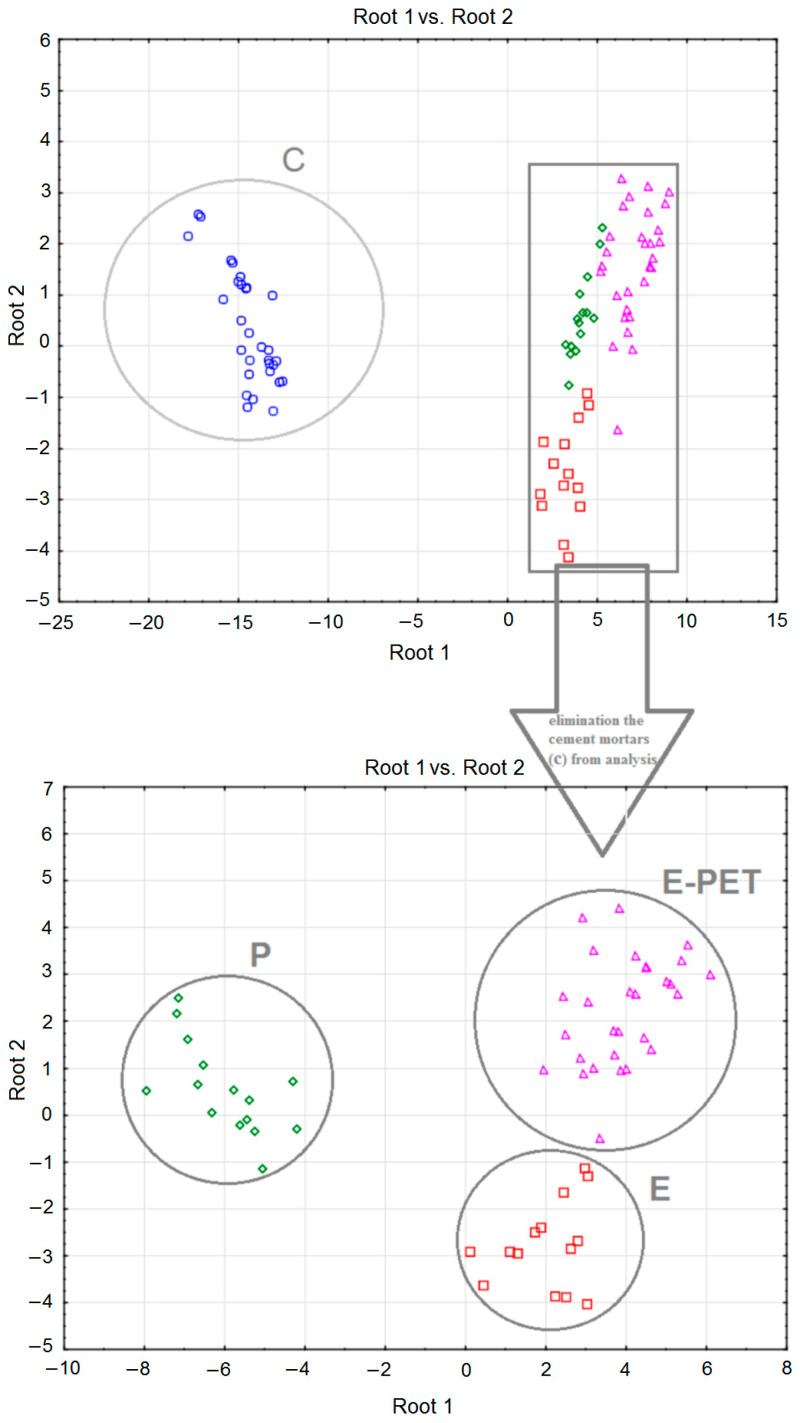
Scatterplots of discriminant functions.

Figure 5 shows the scatter diagrams obtained for the discriminant functions, taking into account those with cement mortars (upper part of the figure) and those without cement mortars (lower part of the figure) in the analysis. Distinctive clusters of different types of mortar are marked. This figure confirms the analysis of the results presented in Table 5, Table 6 and Table 7.

In order to achieve one of the main goals of discriminant analysis, i.e., the classification of new cases, the Statistica program generated a table with so-called classification function coefficients (Table 8). It should be emphasized that classification functions are not the same as the discriminant functions. They are calculated for each group (type of binder) and can be used directly to classify cases (new mortar designs). A case with the highest classification value for a given group may be classified into that group.

Based on the coefficients listed in Table 8, it is possible to create four linear classification functions K1, K2, K3, and K4, which take the form of the relationship (3)–(6):

(3) K1=−370.081−0.523·Fs−0.986·Cs+296.214·Bd+21.846·Abs

(4) K2=−280.149+8.060·Fs−0.716·Cs+206.263·Bd+5.342·Abs

(5) K3=−320.095+10.692·Fs−1.446·Cs+223.188·Bd+5.200·Abs

(6) K4=−336.198+13.101·Fs−1.509·Cs+197.176·Bd+4.319·Abs

The following designations were adopted: *Fs*—Flexural strength, *Cs*—Compressive strength, *Bd*—Bulk density, *Abs*—Absorptivity. 

**Figure 6:**
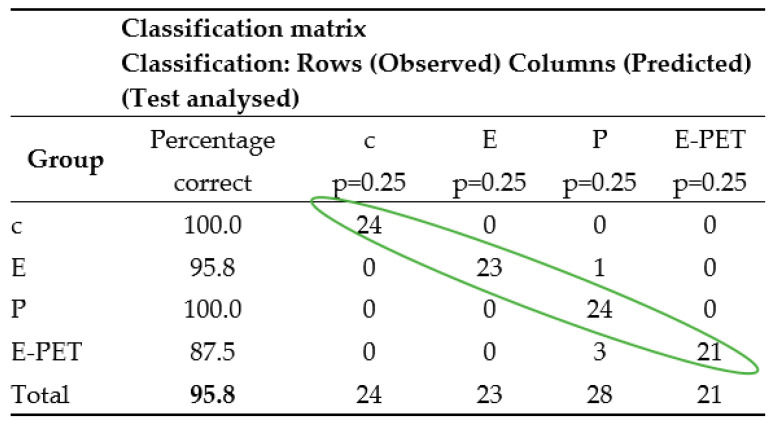
Classification matrix—training group.

In order to decide to what extent the currently determined classification functions allow us to predict whether cases belong to a group, a classification matrix should be generated which shows the number of correctly classified cases (the diagonal marked in the table in Figure 6) and those that have been incorrectly classified.

The correctness of the classification process for the training set was checked on the basis of the data (classification matrix) contained in the table in Figure 6. On its basis, it can be concluded that 95.8% of the cases (from the set of 96) were correctly classified, including cement and polyester mortars, with 100% correct.

The aim of the discrimination process (classification, teaching with the teacher) is to build a rule that can assign new objects as accurately as possible to known (based on the training set) classes.

**Figure 7:**
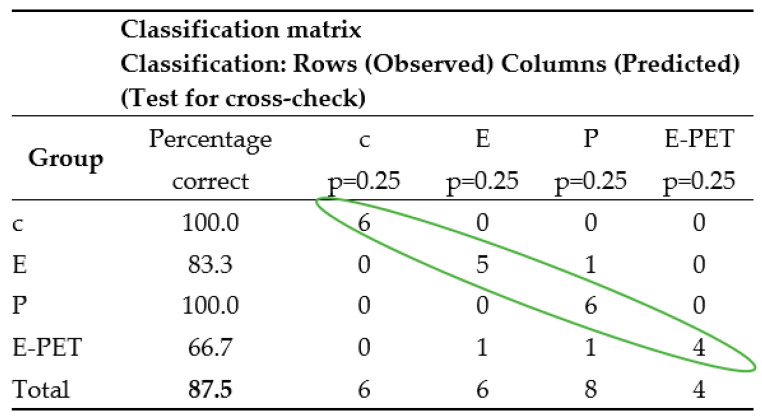
Classification matrix—test group.

Step 2—Testing. The set of test data (24 cases marked in the Stage column with the Test label, which were not previously used to calculate the coefficients of discriminant functions) was used to check the correctness of the classifier, i.e., to assess the correctness of the prognostic functions of the designated classification functions (3)–(6). The results obtained are summarized in the table shown in Figure 7. Correctly classified test data are marked on the diagonal of this table. Based on the results recorded in the table in Figure 7, it can be concluded that the created model allows, in almost 88% of cases, the correct prediction of the type of mortar based on its properties. It can also be stated that, similarly to step 1 (learning), cement and polyester mortars were classified with 100% correct.

The data in Figure 6 and Figure 7 show that the average percentage of correctly classified mortars is slightly higher for the training set (95.8%) than for the test set (87.5%), which is consistent with the results of other researchers.

**Figure 8:**
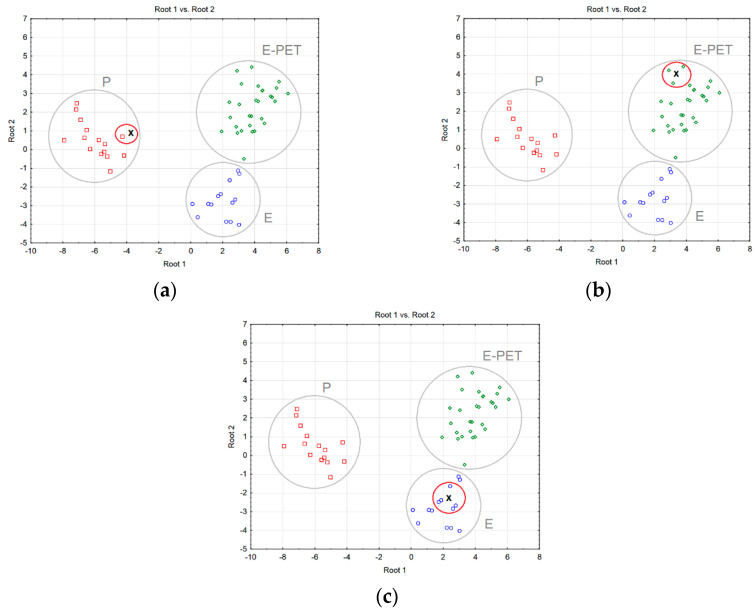
Selection of the mortar composition using the nearest-neighbor method using data from: (**a**) one neighbor, (**b**) three neighbors, (**c**) five neighbors.

Step 3—Classification of new cases. If we assume that the mortar should have specific values of flexural and compressive strength, as well as water absorption and volumetric density, then, based on the classification functions, it will be possible to determine its membership of a given class, and then select its qualitative and quantitative composition. If the class denoting the type of binder has been established, the quantitative composition of the composite is determined on the basis of the composition of the nearest objects adjacent to the designed mortar sample (Figure 8).

By analyzing the scatter plots of discriminant functions to determine, e.g., the amount of binder or hardener for a given mortar, we can use the data of one (Figure 8a), three (Figure 8b) or five neighbors (Figure 8c). In the last two cases, the average of the input parameter values is calculated.

### 3.3. Decision Trees

Another algorithm for searching for patterns that characterize the relationships between the data presented in Figure 3 was classification trees. In this case, the classifier is represented by a binary tree whose nodes contain questions with the value of a specific feature and leaves contain class ratings.

**Figure 9:**
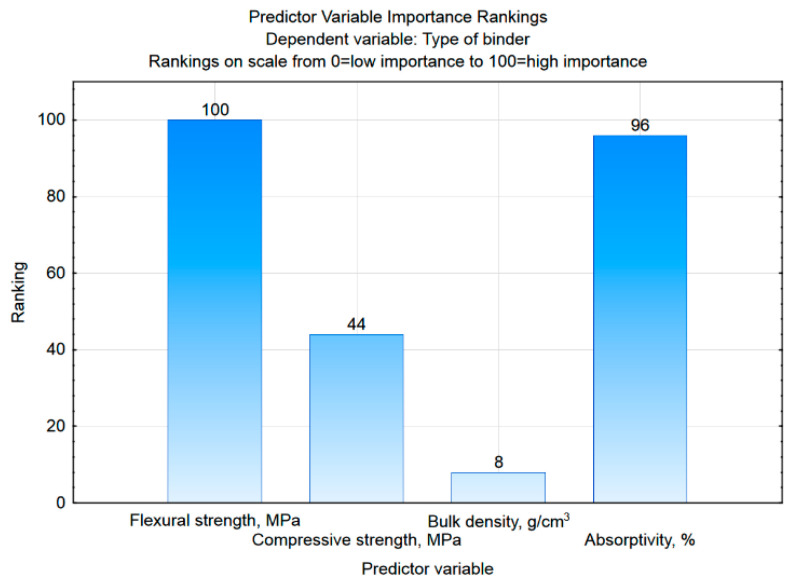
The results of the assessment of the importance of predictors in the classification tree method.

In the case of the studies described in this article, 96 randomly selected mortar samples (marked in the Stage column with the Training label) constituted the training sample used in the tree construction stage, while 24 cases (marked as Test) formed a test group that was used to evaluate the constructed tree. The analysis was started by generating a graph containing a ranking of predictors (Figure 9), which allows one to assess the accuracy of all four variables, i.e., properties that are the results of laboratory tests.

**Figure 10:**
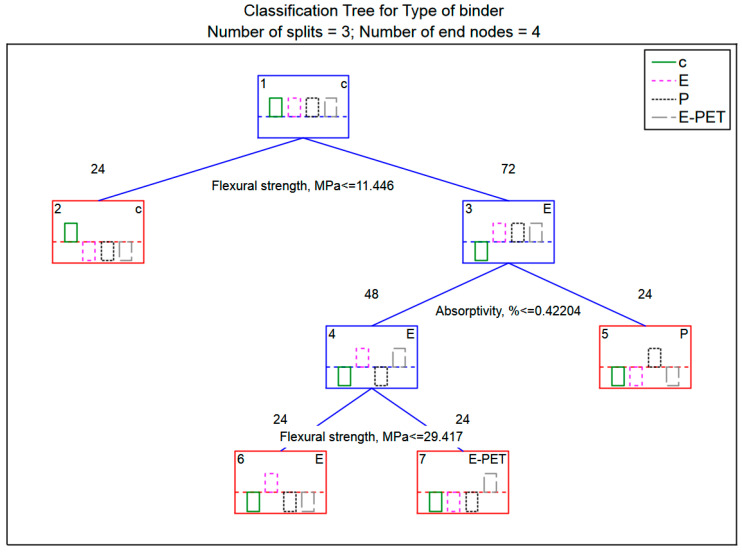
Decision tree.

Based on Figure 9, it can be assessed that the flexural strength and absorptivity are of the highest and almost equal importance (ranking at the level of 100 and 96, respectively), with much lower compressive strength and bulk density the lowest. Such results are consistent with those presented in Table 6 for the first discriminant function, which is characterized by the highest absolute values of the standardized coefficients, which occur precisely for flexural strength and absorptivity. Figure 10 shows the structure of the generated classification tree. It is characterized by the number of divisions (decision nodes) at level 3, i.e., the number of questions generated by the system.

The decision tree presented in Figure 10 can be changed into a set of classification rules. In this case, four rules are distinguished:

Rule 1: if flexural strength ≤ 11.446 MPa then c;
Rule 2: if flexural strength ≥ 11.446 MPa and absorptivity ≥ 0.42204% then P;
Rule 3: if absorptivity ≤ 0.42204% and flexural strength ≥ 29.417 MPa then E-PET;
Rule 4: if absorptivity ≤ 0.42204% and flexural strength ≥ 11.446 MPa and flexural strength ≤ 29.417 MPa then E.

The tree has four end nodes (decision tree “leaves”) that indicate the type of group identified. It can be observed that cement mortars form a well-insulated set, because the shortest decision path leads to this “leaf”, while to the remaining ones it is much longer. Flexural strength turned out to be the most important decision-making attribute, as this feature is tested in the tree root. Subsequently, water absorption value is tested. The other two features (compressive strength and bulk density) turned out to be unnecessary during the decision tree analysis and do not appear in the constructed classification rules. In this case, for the purpose of correct classification, these properties do not need to be determined in the laboratory, which can significantly speed up research work when designing new composites. However, because the database is constantly being updated with new cases that it is planned will be added to the database, the values of all properties should be marked.

In these studies, the correctness of the classification is at a very high level, as evidenced by the results of the classification efficiency of the generated decision tree presented in Table 9 for the training data set and Table 10 for the test data set. In both cases, the 96 samples making up the training set and all 24 samples that make up the test set were correctly classified (the correct classification is 100%).

### 3.4. Cluster Analysis

Cluster analysis, which uses the agglomeration method to form clusters, requires calculations to be made on data recorded in the compared scale. When using measures of discrepancy or distance between objects in the process of creating clusters, the lack of a uniform scale results in the fact that the distance is greatly influenced by the differences between the dimensions in which the data are recorded. The tested properties of mortars have different types of scales (Figure 4); therefore, before starting the analysis, the data were standardized (using the Standardize command, available in the Data menu) such that each variable has an average of 0 and a standard deviation of 1. Thus, the variables that are used to calculate the distance between objects have comparable values, which prevents the analysis from being biased by the different values of the measured parameters and prevents reliance mainly on those dimensions that have the largest range of values.

The conducted analysis aimed to find an answer to the following question. Is it possible to group the mortars covered by the study on the basis of the data from the table presented in Figure 3, that is, do these mortars form “natural” clusters that can be described in a meaningful way?

In order to create clusters of objects (cases, mortars) on the basis of various characteristics (variables, properties), an agglomeration analysis was performed on the collected data. The Agglomeration method list is set to weighted average connections—the calculations take into account the size of the relevant clusters (i.e., the number of objects they contain) as weight. This method should be used when it is suspected that the cluster sizes are clearly unequal. In this way, information was obtained on whether the properties of mortars (variable) form natural aggregates.

The tree grouping method connects successively objects of increasing dissimilarity or distance. There are different ways to calculate the distances. The most direct way to calculate distance is to take k variables as dimensions that make up a k-dimensional space. The analysis used the City Distance (Manhattan) as a measure of distance.

**Figure 11:**
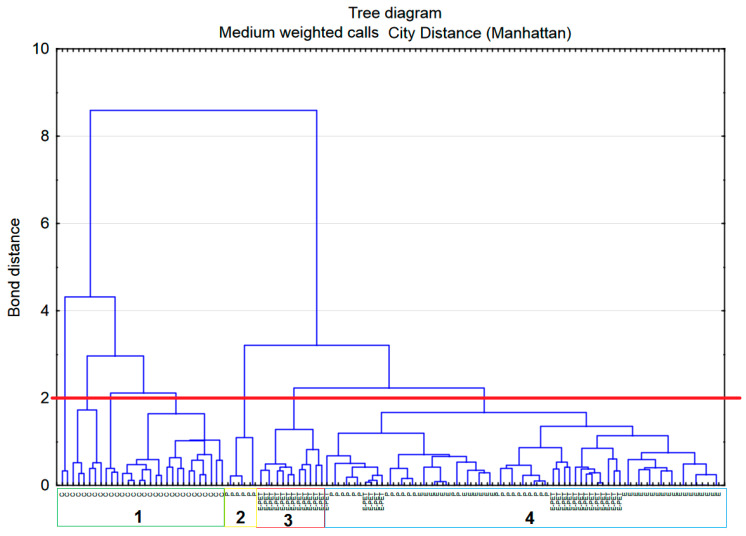
Dendrogram obtained for the four types of mortars.

The resulting dendrogram (Figure 11) starts at the bottom of the figure, where each one-piece sample (each type of mortar) is its own cluster. Moving to the top of the dendrogram, mortars that are “close” are combined into clusters. Each node in the diagram above represents a combination of two or more clusters, and the position of the nodes on the axis represents the distance at which the clusters were formed. The clustering process is complete when an agglomerated cluster containing all of the elements is formed.

The number of clusters depends on the level of intersection of the tree. A cut-off point at a bond distance of 8 divides into two clusters. From the left side we can read that the first of these groups consists of only one type of mortar, cement (marked c). The other types of mortars (P, E-PET, and E) were included in the second group. This division results from the differences in the absorptivity of the mortars; for cement mortars this feature is much higher than for the others. At a bonding distance of approximately 4, the cluster marked c has split into two. The cutoff point at a bond distance of 2 appears to be the most advantageous. There are seven clusters in this case—four within cement mortars, one formed by polyester mortars, one composed of E-PET mortars and one with all epoxy mortars, but also polyester and E-PET mortars. The clusters assigned to cement mortars were treated as one; therefore, in further analyses, the number of clusters was assumed to be four (as indicated in Figure 11).

**Figure 12:**
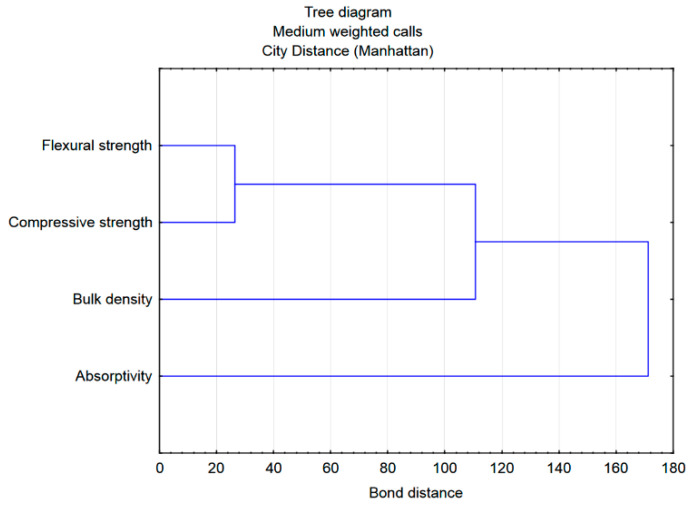
Dendrogram obtained for the properties tested of mortars.

**Figure 13:**
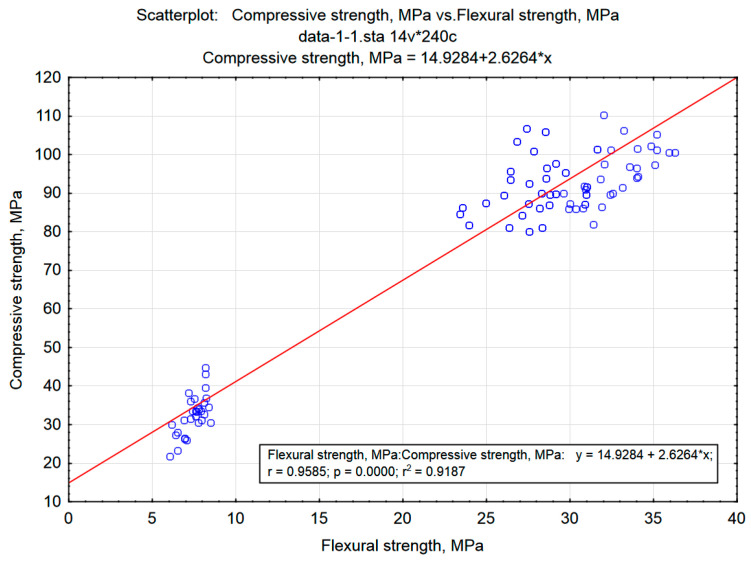
Scatterplot: Compressive strength vs. Flexural strength.

A reflection of this division is a tree diagram created for the same data, but taking into account grouping by columns (variables, i.e., mortar properties) (Figure 12). Here, the properties of the mortars formed the four clusters. With a cut-off at a distance of approximately 170, a division into a cluster formed by absorptivity and a group (cluster) of other properties arises. The latter, at a distance of approximately 110, is divided into the next two clusters: bulk density and strength parameters. At a distance of approximately 25, a division into flexural and compressive strength can be distinguished. The assessment of the bonding distance (very small) between the strength variables shows that these variables are located close to each other in the feature space, which shows that they are correlated. This fact is confirmed by the location of the points on the scatter plot shown in Figure 13 and the results of the variance analysis (e.g., similar values of the F parameter) presented in Table 11.

Cluster analysis allows for grouping mortars also by another method: k-means grouping. The goal of the k-means algorithm is to find the optimal division of objects into k clusters. The procedure involves moving objects from one cluster to another to minimize the variance within the clusters and maximize the variance between clusters. When analyzing the tree diagram, four main clusters were distinguished. Thus, in the analyses performed using the k-means method, just such a number of clusters was assumed.

In the Statistica Analysis of Variance module, the variance is compared to the variance within groups. On this basis, it can be concluded whether the means of a given variable in the groups differ significantly. Displaying the Analysis of Variance window allows us to compare the means for each mortar property in groups (mortar clusters). The results of this analysis are presented in Table 11. Taking into account the values (and significance levels) of the F values, the Absoptivity, Compressive and Flexural strength variables constitute the main criteria for assigning mortars to clusters.

**Figure 14:**
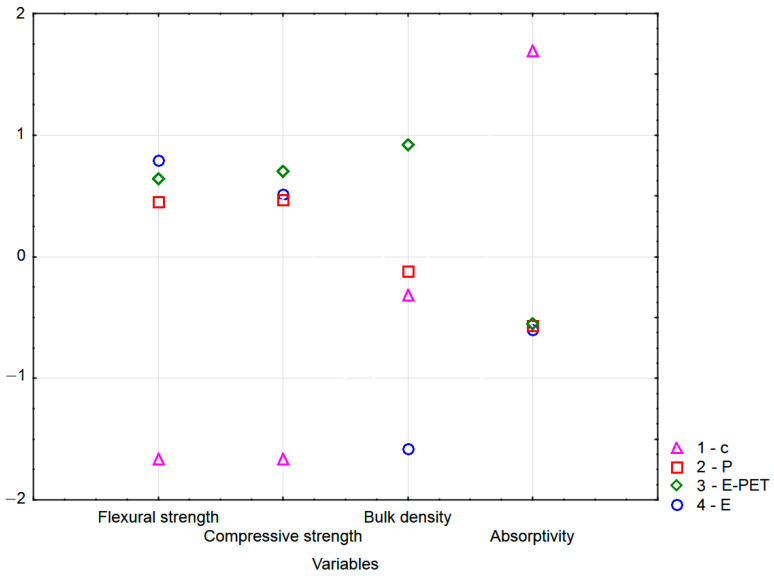
Graph of the normalized mean values of four properties designated for each focus.

The graphical summary of this analysis is a plot of the means of each cluster (Figure 14).

By analyzing the average diagrams of each cluster included in Figure 14, it can be concluded that a cluster containing only cement mortars definitely stands out from the others, both in terms of strength and water absorption parameters. In turn, in cluster 2, where only polyester mortars are located, the largest differences are in volume density. There are also differences in the average strength parameters of the resin mortars. It should be emphasized that these conclusions are appropriate for the results of the discussion presented above, i.e., when discussing Figure 4.

## 4. Conclusions

In the context of the research and analyses carried out, it can be stated that:

- It was found that the modification of cement and resin mortars, consisting in partial replacement of aggregate with PE waste agglomerate, allows for obtaining mortars with very good strength parameters;
- It has been observed that the inclusion of waste into the composition of the mortar does not significantly change the bulk density and water absorption;
- It has been shown that simultaneous modification of mortar composition by glycolysate formed on the basis of PET waste and PE waste agglomerate has a particularly beneficial effect on the properties and cost of obtaining epoxy mortars;
- The variety and multiplicity of data mining methods make it difficult for potential users to choose the methods that are most appropriate to their data analysis needs. When different methods give similar results, the strength of the conclusions drawn increases;
- The three methods of data mining used led to similar results; however, in the discriminant analysis, the degree of correctness of the classification is much lower than in the other two methods;
- On the other hand, unlike decision trees and cluster analysis, discriminant analysis allows one to build classification functions that make it possible to predict the composition of mortars for a larger number of desired properties;
- For decision trees and cluster analysis, the ease of understanding and interpretation of the visualized results is important. The possibility of creating logical decision rules on their basis may be easier to interpret than explaining the meaning of the coefficients of the generated classification functions obtained after applying discriminant analysis;
- The developed methodology for creating classification systems can be used in research on other composites.

## Figures and Tables

**Table 1:** Selected properties of cement.

			Cement Properties		
Cement Type	Compressive Strength (28 Day), MPa	Loss on Ignition, %	Content of Sulphates, %	Chlorides Content, %	Beginning of Setting Time, min
CEM I 42,5 R	≥42.5, ≤62.5	≤5	≤4.0	≤0.10	≥60

**Table 2:** Selected properties of resin binders.

			Resin Properties		
Resin Type	Density, g/cm^3^	Viscosity 25 °C, mPa s	Molecular Weight, g/mol	Epoxy Count LE, mol/100 g	Acid Numer LK, mg KOH/g
Epidian 5	1.17	30,000	450	0.49	-
Polimal 109	1.10–1.16	350	-	-	32

**Table 3:** Selected properties of hardeners used in research.

	Hardener Properties			
Hardener Type	Density, g/cm^3^	Gel Time, min	Viscosity 25 °C, mPa s	Amine Number, mg KOH/g
Z-1	0.978–0.983	-	20–30	Min. 1100
Metox-50	1.169–1.175	24–26	-	-

**Table 4:** Assessment of the contribution of individual variables to the overall discrimination of mortars.

	Summary of the Discriminant Function Analysis: N var. in the Model: 4; Grouping: Type of Binder (4 Groups) Wilks’ Lambda: 0.00306 Approximately F (12.3) = 197.6 *p* < 0.0000					
N = 120	Wilks’ Lambda	Partial Wilks	F Removed (3.113)	*p*	Toler.	1-Toler. (R^2^)
Flexural strength	0.017	0.176	176.55	0.0000	0.502	0.498
Compressive strength	0.006	0.507	36.67	0.0000	0.384	0.616
Bulk density	0.004	0.692	16.73	0.0000	0.652	0.348
Absorptivity	0.006	0.529	33.95	0.0000	0.911	0.089

**Table 5:** Summary of the results of the Chi-square test.

	Chi-Square Tests of the Following Roots					
Roots Removed	Eigenvalue	Canonical R	Wilks’ Lambda	Chi-Square	df	*p*
0	74.083	0.993	0.003	665.876	12	0.0000
1	2.376	0.839	0.229	169.237	6	0.0000
2	0.290	0.474	0.775	29.309	2	0.0000

**Table 6:** List of coefficients of standardized canonical discriminant functions.

	Standardized Coefficients for Canonical Variables		
Variable	Root 1	Root 2	Root 3
Flexural strength	0.926	1.064	−0.004
Compressive strength	−0.109	−1.269	−0.794
Bulk density	−0.422	0.288	1.027
Absorptivity	−0.612	0.335	−0.569
Eigenvalue	74.083	2.376	0.290
Cum. prop.	0.965	0.996	1.000

**Table 7:** Average canonical variables of the discriminant functions.

	Average Canonical Variables		
Group	Root 1	Root 2	Root 3
c	−14.454	0.364	−0.084
E	3.259	−2.505	−0.183
P	4.142	0.578	0.857
E-PET	7.053	1.564	−0.590

**Table 8:** Parameters of the classification functions.

	Classification Functions; Grouping Variable: Type of Binder			
Variable	c *p* = 0.25	E *p* = 0.25	P *p* = 0.25	E-PET *p* = 0.25
Flexural strength	−0.523	8.060	10.692	13.101
Compressive strength	−0.986	−0.716	−1.446	−1.509
Bulk density	296.214	206.263	223.188	197.176
Absorptivity	21.846	5.342	5.200	4.319
Constant (free term)	−370.081	−280.149	−320.095	−336.198

**Table 9:** Results of incorrect classifications for the training sample.

	Learning Sample Misclassification Matrix Predicted (Row) × Observed (Column) Matrix Learning Sample N = 96			
Class	Class c	Class E	Class P	Class E-PET
c		0	0	0
E	0		0	0
P	0	0		0
E-PET	0	0	0	

**Table 10:** Results of incorrect classifications for the test group sample.

	Test Sample Misclassification Matrix Predicted (Row) × Observed (Column) Matrix CV Cost = 0; s.d. CV Cost = 0			
Class	Class c	Class E	Class P	Class E-PET
c		0	0	0
E	0		0	0
P	0	0		0
E-PET	0	0	0	

**Table 11:** Results of the analysis of variance.

Variable	Between SS	df	Int. SS	df	F	Relevant *p*
Flexural strength	111.63	3	7.37	116	585.71	0.00000
Compressive strength	112.05	3	6.95	116	623.41	0.00000
Bulk density	53.34	3	65.66	116	31.41	0.00000
Absorptivity	114.82	3	4.18	116	1061.05	0.00000

## Data Availability

Not applicable.

## References

[1] da Silva T.R., de Azevedo A.R.G., Cecchin D., Marvila M.T., Amran M., Fediuk R., Vatin N., Karelina M., Klyuev S., Szelag M. (2021). Application of Plastic Wastes in Construction Materials: A Review Using the Concept of Life-Cycle Assessment in the Context of Recent Research for Future Perspectives. Materials.

[2] Beghetto V., Sole R., Buranello C., Al-Abkal M., Facchin M. (2021). Recent Advancements in Plastic Packaging Recycling: A Mini-Review. Materials.

[3] Shang M., Li H., Ahmad A., Ahmad W., Ostrowski K.A., Aslam F., Joyklad P., Majka T.M. (2022). Predicting the Mechanical Properties of RCA-Based Concrete Using Supervised Machine Learning Algorithms. Materials.

[4] Belmokaddem M., Mahi A., Senhadji Y., Pekmezci B.Y. (2020). Mechanical and physical properties and morphology of concrete containing plastic waste as aggregate. Constr. Build. Mater..

[5] Jacob-Vaillancourt C.L. (2018). Characterization of concrete composites with recycled plastic aggregates from postconsumer material streams. Constr. Build. Mater..

[6] Assaad J.J., Khatib J.M., Ghanem R. (2022). Bond to Bar Reinforcement of PET-Modified Concrete Containing Natural or Recycled Coarse Aggregates. Environments.

[7] Li G., Zhang L., Zhao F., Tang J. (2020). Acoustic Emission Characteristics and Damage Mechanisms Investigation of Basalt Fiber Concrete with Recycled Aggregate. Materials.

[8] Thorneycroft J., Orr J., Savoikar P., Ball R.J. (2018). Performance of structural concrete with recycled plastic waste as a partial replacement for sand. Constr. Build. Mater..

[9] Jones H., Saffar F., Koutsos V., Ray D. (2021). Polyolefins and Polyethylene Terephthalate Package Wastes: Recycling and Use in Composites. Energies.

[10] Awoyera P.O., Adesina A. (2020). Plastic wastes to construction products: Status, limitations and future perspective. Case Stud. Constr. Mater..

[11] Meza A., Pujadas P., Meza L.M., Pardo-Bosch F., López-Carreño R.D. (2021). Mechanical Optimization of Concrete with Recycled PET Fibres Based on a Statistical-Experimental Study. Materials.

[12] Poonyakan A., Rachakornkij M., Wecharatana M., Smittakorn W. (2018). Potential Use of PlasticWastes for Low Thermal Conductivity Concrete. Materials.

[13] Chen Y., Xu L., Xuan W., Zhou Z. (2019). Experimental study on four-point cyclic bending behaviours of concrete with high density polyethylene granules. Constr. Build. Mater..

[14] Dębska B., Lichołai L. (2018). Long-term chemical resistance of ecological epoxy polymer composites. J. Ecol. Eng..

[15] STATISTICA 12, StatSoft Polska Sp. z. o. o. www.statsoft.pl.

[16] Ahmad M., Hu J.-L., Ahmad F., Tang X.-W., Amjad M., Iqbal M.J., Asim M., Farooq A. (2021). Supervised Learning Methods for Modeling Concrete Compressive Strength Prediction at High Temperature. Materials.

[17] Ahmad A., Farooq F., Niewiadomski P., Ostrowski K., Akbar A., Aslam F., Alyousef R. (2021). Prediction of Compressive Strength of Fly Ash Based Concrete Using Individual and Ensemble Algorithm. Materials.

[18] Chaabene W.B., Flah M., Nehdi M.L. (2020). Machine learning prediction of mechanical properties of concrete: Critical review. Constr. Build. Mater..

[19] Rathakrishnan V., Beddu S.B., Ahmed A.N. (2022). Predicting compressive strength of high-performance concrete with high volume ground granulated blast-furnace slag replacement using boosting machine learning algorithms. Sci. Rep..

[20] Czarnecki S., Sadowski Ł., Hoła J. (2021). Evaluation of interlayer bonding in layered composites based on non-destructive measurements and machine learning: Comparative analysis of selected learning algorithms. Autom. Constr..

[21] Tran V.Q., Mai H.-V.T., Nguyen T.-A., Ly H.-B. (2021). Investigation of ANN architecture for predicting the compressive strength of concrete containing GGBFS. PLoS ONE.

[22] Malazdrewicz S., Sadowski Ł. (2021). An intelligent model for the prediction of the depth of the wear of cementitious composite modified with high-calcium fly ash. Compos. Struct..

[23] Kang M.-C., Yoo D.-Y., Gupta R. (2021). Machine learning-based prediction for compressive and flexural strengths of steel fiber-reinforced concrete. Constr. Build. Mater..

[24] Wang F., Wang Q., Nie F., Li Z., Yu W., Ren F. (2020). A linear multivariate binary decision tree classifier based on K-means splitting. Pattern Recognit..

[25] Behnood A., Golafshani E.M. (2020). Machine learning study of the mechanical properties of concretes containing waste foundry sand. Constr. Build. Mater..

[26] Behnood A., Behnood V., Gharehveran M.M., Alyamac K.E. (2017). Prediction of the compressive strength of normal and high-performance concretes using M5P model tree algorithm. Constr. Build. Mater..

[27] Arora S., Singh B., Bhardwaj B. (2019). Strength performance of recycled aggregate concretes containing mineral admixtures and their performance prediction through various modeling techniques. J. Build. Eng..

[28] Madra A., Adrien J., Breitkopf P., Maire E., Trochu F. (2017). A clustering method for analysis of morphology of short natural fibers in composites based on X-ray microtomography. Compos. Part A.

[29] Jain D., Mukherjee A., Bera T.K. (2018). A novel characterization method of fiber reinforced polymers with clustered microstructures for time dependent mass transfer. Sci. Eng. Compos. Mater..

[30] Kılınçarslan Ş., İnce E.Y., Tuncay E.B., Yağmurlu F. (2018). Clustering Analysis of Normal Strength Concretes Produced with Different Aggregate Types. Open Chem..

[31] (2016). Cement Testing Methods—Part 1: Determination of Strength.

[32] Vítková G., Prokeš L., Novotný K., Pořízk P., Novotný J., Všianský D., Čelko L., Kaiser J. (2014). Comparative study on fast classification of brick samples by combination of principal component analysis and linear discriminant analysis using stand-off and table-top laser-induced breakdown spectroscopy. Spectrochim. Acta Part B At. Spectrosc..

[33] Sutton C.D., Pfefferman D. (2010). Classification and regression trees, bagging and boosting. Handbook of Statistics.

[34] Koronacki J., Ćwik J. (2008). Statystyczne Systemy Uczące Się.

[35] Almuallim H., Kaneda S., Akiba Y. (2002). Development and applications of decision trees. Expert. Syst..

[36] Kotsiantis S.B. (2007). Supervised machine learning: A review of classification techniques. Informatica.

[37] StatSoft (2006). Elektroniczny Podręcznik Statystyki PL, Krakow. http://www.statsoft.pl/textbook/stathome.html.

[38] Maciejewska M. (2012). Analiza Danych w Czujnikowych Pomiarach Zanieczyszczeń Powietrza.

